# The Complementary Roles of Chloroplast Cyclic Electron Transport and Mitochondrial Alternative Oxidase to Ensure Photosynthetic Performance

**DOI:** 10.3389/fpls.2021.748204

**Published:** 2021-09-28

**Authors:** Avesh Chadee, Nicole A. Alber, Keshav Dahal, Greg C. Vanlerberghe

**Affiliations:** ^1^Department of Biological Sciences, and Department of Cell and Systems Biology, University of Toronto Scarborough, Toronto, ON, Canada; ^2^Fredericton Research and Development Centre, Agriculture and Agri-Food Canada, Fredericton, NB, Canada

**Keywords:** ATP/NADPH, cyclic electron transport, energy and carbon balance, alternative oxidase, malate valve, mitochondrial electron transport, proton gradient regulation5/PGR5-like photosynthetic phenotype1, reactive oxygen species

## Abstract

Chloroplasts use light energy and a linear electron transport (LET) pathway for the coupled generation of NADPH and ATP. It is widely accepted that the production ratio of ATP to NADPH is usually less than required to fulfill the energetic needs of the chloroplast. Left uncorrected, this would quickly result in an over-reduction of the stromal pyridine nucleotide pool (i.e., high NADPH/NADP^+^ ratio) and under-energization of the stromal adenine nucleotide pool (i.e., low ATP/ADP ratio). These imbalances could cause metabolic bottlenecks, as well as increased generation of damaging reactive oxygen species. Chloroplast cyclic electron transport (CET) and the chloroplast malate valve could each act to prevent stromal over-reduction, albeit in distinct ways. CET avoids the NADPH production associated with LET, while the malate valve consumes the NADPH associated with LET. CET could operate by one of two different pathways, depending upon the chloroplast ATP demand. The NADH dehydrogenase-like pathway yields a higher ATP return per electron flux than the pathway involving PROTON GRADIENT REGULATION5 (PGR5) and PGR5-LIKE PHOTOSYNTHETIC PHENOTYPE1 (PGRL1). Similarly, the malate valve could couple with one of two different mitochondrial electron transport pathways, depending upon the cytosolic ATP demand. The cytochrome pathway yields a higher ATP return per electron flux than the alternative oxidase (AOX) pathway. In both *Arabidopsis thaliana* and *Chlamydomonas reinhardtii*, PGR5/PGRL1 pathway mutants have increased amounts of AOX, suggesting complementary roles for these two lesser-ATP yielding mechanisms of preventing stromal over-reduction. These two pathways may become most relevant under environmental stress conditions that lower the ATP demands for carbon fixation and carbohydrate export.

## Introduction

Research over many years has established that chloroplasts and mitochondria act in a coordinated manner during photosynthesis (Krömer, 1995; Raghavendra and Padmasree, 2003). For example, mitochondria oxidize the glycine associated with photorespiration and likely supply the bulk of the cytosolic ATP required for sucrose synthesis and export. More generally, optimal photosynthesis requires the maintenance of cellular energy and carbon balance (Paul and Foyer, 2001; Stitt et al., 2010; Kramer and Evans, 2011). Changes in the availability of light, CO_2_, and other environmental parameters can disrupt this balance. Hence, specific respiratory activities may aid in maintaining energy and carbon balance in the light, thereby directly optimizing photosynthesis. Early genetic studies in the green alga *Chlamydomonas reinhardtii* illustrated the complexity and potential plasticity of such organelle interactions (Lemaire et al., 1988; Cardol et al., 2009).

This review will briefly introduce the concepts of energy and carbon balance during C_3_ photosynthesis and then focus on two pathways of electron flow; cyclic electron transport (CET) in the chloroplast and alternative oxidase (AOX) respiration in the mitochondrion. The coordinated activity of these pathways may have a central role in maintaining energy and carbon balance during photosynthesis and in response to changing environmental conditions.

## The Need to Maintain Energy and Carbon Balance During Photosynthesis

During C_3_ photosynthesis in leaf mesophyll cells, a light-driven chloroplast electron transport chain (cETC) in the thylakoid membrane generates energy intermediates (ATP and NADPH) that are then utilized by a CO_2_-dependent carbon fixation process in the chloroplast stroma [the Calvin-Benson (CB) cycle] to produce triose phosphates (TP; Stitt et al., 2010). Two major challenges for the chloroplast during this process are (1) to balance the overall rate of light-driven production of energy intermediates with their rate of consumption, principally by the CB cycle and (2) to match the production ratio of ATP to NADPH with its consumption ratio by chloroplast metabolism (Kramer and Evans, 2011). These two distinct aspects of energy balance are closely related. For example, if the production ratio of ATP to NADPH was too low to meet demand, then the shortfall of ATP would slow the CB cycle, the major consumer of energy intermediates in the chloroplast. This in turn would generate an additional imbalance between light energy absorption and use.

In linear electron transport (LET), photosystem II (PSII) uses light energy to extract electrons from water and pass these through the plastoquinone (PQ) pool, the cytochrome *b_6_f* (cyt *b_6_f*) complex, and plastocyanin to photosystem I (PSI). PSI then uses additional light energy to pass the electrons through ferredoxin to reduce NADP^+^ to NADPH. During LET, the splitting of water and PQ oxidation by the cyt *b_6_f* complex each contributes to generating a *trans*-thylakoid proton gradient (ΔpH). The proton motive force (*pmf*) associated with this gradient is then used by the chloroplast ATP synthase (cATP synthase) to generate ATP from ADP and inorganic phosphate (P_i_) by photophosphorylation. However, it is widely acknowledged that the production ratio of ATP to NADPH generated by LET is less than required by stromal metabolism (mainly carbon fixation and photorespiration), hence necessitating mechanism(s) to boost this ratio. Complicating this further, the ATP/NADPH balance required in the stroma to satisfy photorespiration is higher than needed to satisfy carbon fixation, meaning that the optimal ATP/NADPH production ratio will change if the ratio of Rubisco carboxylation to oxygenation changes (Backhausen and Scheibe, 1999; Noctor and Foyer, 2000; Foyer et al., 2012; Walker et al., 2016).

One means by which chloroplasts maintain energy balance during photosynthesis is to make use of additional secondary pathways of electron flow that support ΔpH generation, and hence net ATP synthesis, but not the net synthesis of NADPH. In C_3_ plants, potential additional pathways include the malate valve, the Mehler reaction, the plastid terminal oxidase, and two different pathways of CET around PSI. In the CET pathways, electrons in ferredoxin are cycled back through the PQ pool and cyt *b_6_f* complex (Heber and Walker, 1992; Johnson, 2011; Yamori and Shikanai, 2016). In the malate valve, stromal NADPH is consumed by the reduction of oxaloacetate (OAA) to malate, which is then exported to the cytosol in exchange for OAA (Selinski and Scheibe, 2018). As described later, this process has important links to mitochondrial respiration. In the Mehler reaction, an electron from PSI is transferred to oxygen generating superoxide, the controlled detoxification of which then consumes stromal NADPH (Asada, 2006). Finally, plastid terminal oxidase couples PQ oxidation with the reduction of oxygen to water (Nawrocki et al., 2015). Another recently described pathway uses the stromal enzyme phosphoglycerate dehydrogenase to indirectly transfer electrons from the NADPH to NADH pool (Höhner et al., 2021).

Another means by which chloroplasts maintain energy balance during photosynthesis is to use the thylakoid ΔpH buildup generated by electron flow (both LET and the secondary pathways) as a means to feedback-regulate LET. This provides photo-protection by preventing the over-reduction of cETC components. The two main ΔpH-dependent photo-protective processes are “non-photochemical quenching” (NPQ) mechanisms and “photosynthetic control.” NPQ mechanisms increase the fraction of absorbed light energy at PSII that is dissipated as heat rather than supporting photochemical PQ reduction (Wobbe et al., 2016; Murchie and Ruban, 2020). This protects PSII from singlet oxygen-induced photo-damage and slows the rate of electron flow into the cETC. Photosynthetic control refers to when lumen acidification inhibits the rate of PQ oxidation by the cyt *b_6_f* complex, hence slowing the rate of electron flow toward PSI (Foyer et al., 1990; Tikhonov, 2013). This prevents the over-reduction of electron carriers on the PSI acceptor side, which can otherwise result in PSI photo-damage (Wobbe et al., 2016).

A third major challenge for the chloroplast during photosynthesis relates to carbon balance. Effective plant growth and development require a balance between source and sink activities (Fernie et al., 2020). If source leaf sucrose production in the light outpaces sucrose utilization by the sinks, then phloem transport will slow and source leaf sucrose concentration will rise. A buildup of cytosolic sugar phosphate precursors (at the expense of cytosolic P_i_) will then slow the rate of sucrose synthesis, by still poorly understood feedback mechanisms (Baena-González and Lunn, 2020). The reduction of cytosolic P_i_ may then slow its exchange by the triose phosphate translocator for stromal TP’s. As sugar phosphates then accumulate in the chloroplast, stromal P_i_ will also decline. This may then limit cATP synthase activity and slow photosynthesis (Stitt et al., 2010; Morales et al., 2018; McClain and Sharkey, 2019). Persistent carbon imbalances can also reduce photosynthetic capacity through changes in gene expression (Paul and Foyer, 2001).

Continuing research aims to understand the extent to which each of the secondary pathways of electron flow contribute to the maintenance of energy balance. It seems possible that different pathways will prevail, depending upon their own unique biochemical and molecular characteristics, as well as the particular environmental conditions in which photosynthesis is operating (Backhausen et al., 2000). In quantitative terms, CET and the malate valve (coupled with mitochondrial respiration) are likely to be the most prominent pathways in C_3_ plants (Walker et al., 2020). Hence, these pathways, and their potential interaction with one another to maintain energy balance, are the major subject of this review. The review will also briefly consider how these pathways may contribute to the maintenance of carbon balance during photosynthesis.

## Two Distinct Pathways of Cet Around Photosystem I

CET around PSI acts to increase the ATP/NADPH production ratio of the cETC by avoiding the production of NADPH, while supporting the buildup of ΔpH for ATP synthesis. By balancing the ATP/NADPH ratio in the stroma, CET prevents metabolic bottlenecks that would otherwise slow the CB cycle. A balanced ATP/NADPH ratio allows high CB cycle activity and hence a high capacity to turn over the purine and adenine nucleotide pools. This ensures that the plant can take advantage of conditions when light and CO_2_ are abundant. In addition, while CET is not a net sink for electrons, it does contribute to photo-protection since the buildup of ΔpH can support increases in NPQ at PSII and photosynthetic control at cyt *b_6_f*.

**Figure 1:**
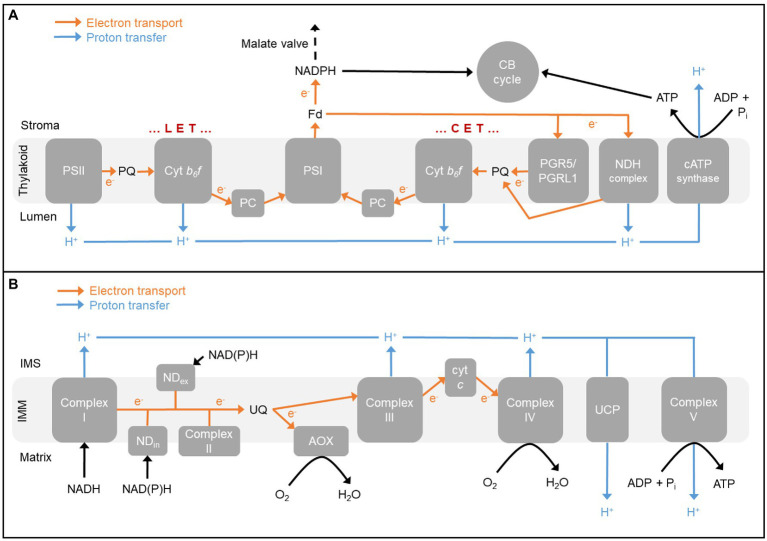
**(A)** The photosynthetic electron transport chain in chloroplast thylakoid membrane. Linear electron transport (LET) involves both photosystems while cyclic electron transport (CET) involves only photosystem I (PSI). Both modes of electron transport contribute to the proton motive force used by chloroplast ATP synthase (cATP synthase) to generate ATP, while only LET contributes to NADPH production. ATP and NADPH support carbon fixation by the Calvin-Benson (CB) cycle. One pathway of CET involves the proton-pumping NADH dehydrogenase-like (NDH) complex, while the other pathway involves the proteins PROTON GRADIENT REGULATION5 (PGR5) and PGR5-LIKE PHOTOSYNTHETIC PHENOTYPE1 (PGRL1). The malate valve can transfer NADPH equivalents from the stroma to cytosol. **(B)** The respiratory electron transport chain in inner mitochondrial membrane (IMM). Complex I, a family of other internal and external alternative dehydrogenases (ND_in_ and ND_ex_, respectively), and Complex II oxidize reducing equivalents. Electrons partition between the cytochrome (cyt) pathway [involving Complex III, cyt *c*, and Complex IV (cyt *c* oxidase)] and alternative oxidase (AOX). The proton motive force generated by electron transport is used by the mitochondrial ATP synthase (Complex V) to generate ATP. The proton gradient can also be dissipated by uncoupling proteins (UCP). Electron flow from reducing equivalents to cyt *c* oxidase is more tightly coupled to the generation of proton motive force (2–3 sites of proton translocation) than is electron flow from reducing equivalents to AOX (0–1 sites of proton translocation). Fd, ferredoxin; IMS, intermembrane space; and PC, plastocyanin.

There are two pathways of CET around PSI in C_3_ plants (Figure 1A). The major pathway involves the PROTON GRADIENT REGULATION5 (PGR5) and PGR5-LIKE PHOTOSYNTHETIC PHENOTYPE1 (PGRL1) proteins (Munekage et al., 2002; DalCorso et al., 2008; Hertle et al., 2013; Rühle et al., 2021). The phenotype of PGR5/PGRL1 pathway mutants is an inability to engage NPQ due to insufficient buildup of ΔpH across the thylakoid membrane. However, the precise molecular function of these proteins in the pathway remains uncertain (Shikanai, 2020). A distinguishing feature of the PGR5/PGRL1 pathway is its inhibition by antimycin A (Munekage et al., 2002; Nishikawa et al., 2012; Sugimoto et al., 2013; Rühle et al., 2021). The second pathway of CET involves an NADH dehydrogenase-like complex (NDH; Joët et al., 2001; Shikanai, 2016; Strand et al., 2017a; Schuller et al., 2019). The NDH complex is composed of at least 29 subunits (*Arabidopsis thaliana*), forms a super-complex with PSI, and is proton-pumping meaning that it is higher ATP yielding (per electron transported) than the PGR5/PGRL1 pathway. Double mutants lacking both CET pathways show severe photosynthetic and growth phenotypes, even under relatively optimal growth conditions, illustrating the essential role of CET in C_3_ plants (Munekage et al., 2004).

While PGR5/PGRL1 is considered the main pathway of CET in C_3_ plants, there is evidence that both pathways contribute to CET and can at least partially compensate for each other’s activity (Joët et al., 2001; Munekage et al., 2004; Munné-Bosch et al., 2005; Wang et al., 2015; Nakano et al., 2019). That said, there is accumulating evidence that the NDH pathway has added importance at lower irradiances, while the PGR5/PGRL1 pathway has added importance at higher irradiances. Since the ratio of photorespiration to carbon fixation typically increases with irradiance, it exaggerates the shortfall of ATP relative to NADPH production by LET. Hence, while the lower capacity (but higher ATP yielding) NDH pathway may be sufficient at low irradiance, the PGR5/PGRL1 pathway may need to make an additional contribution to CET when the ATP/NADPH imbalance is greater, such as at higher irradiance. Fisher et al. (2019) showed that ATP inhibits both CET pathways, with the PGR5/PGRL1 pathway being 2–3 fold more sensitive to such downregulation (i.e., lower half-maximal inhibitory concentration for ATP). This suggests that a moderate shortfall of ATP relative to NADPH production by LET (such as at low irradiance) should first engage the NDH pathway, while a more severe ATP shortfall (such as at high irradiance) would additionally engage the PGR5/PGRL1 pathway. According to this logic, engagement of the PGR5/PGRL1 pathway should require a more severe over-reduction of the stroma than engagement of the NDH pathway.

Studies across multiple species suggest that the NDH pathway is most relevant at lower irradiances (Ueda et al., 2012; Kou et al., 2015; Martín et al., 2015; Yamori et al., 2015). NDH mutants in liverwort (*Marchantia polymorpha*) and rice (*Oryza sativa*) display higher excitation pressure specifically at low irradiance, and the rice mutant shows reduced carbon fixation and growth at low but not high irradiance (Ueda et al., 2012; Yamori et al., 2015). It seems probable that, when photosynthesis is light-limited, the higher ATP-yielding NDH pathway of CET is best suited to balance the ATP/NADPH production ratio of the chloroplast. Studies have also compared the thylakoid proteome of low, medium, and high irradiance-grown plants. In both pea (*Pisum sativum*) and *A. thaliana*, the protein amounts of both CET pathways increased with growth irradiance (Albanese et al., 2018; Flannery et al., 2021). However, only PGR5/PGRL1 pathway components increased substantially in *A. thaliana* between medium and high irradiance. Evidence is also emerging that the CET pathways are controlled, at least in part, by the thiol-based redox regulation systems of the chloroplast. These include the ferredoxin-thioredoxin reductase and NADPH-thioredoxin reductase C systems (Yoshida and Hisabori, 2016a). Accumulating data suggest that low light may preferentially activate the NDH pathway *via* NADPH-thioredoxin reductase C, while high light may preferentially activate the PGR5/PGRL1 pathway *via* ferredoxin-thioredoxin reductase (Nikkanen and Rintamäki, 2019). Overall, multiple lines of evidence suggest that while both CET pathways may increase in activity with increasing irradiance, there may also be a gradual shift in electron partitioning toward the PGR5/PGRL1 path as irradiance increases.

Nonetheless, what is the advantage of having two CET pathways rather than, for example, just a higher capacity NDH pathway? As irradiance increases and/or the leaf concentration of CO_2_ for carbon fixation becomes more limiting, it is advantageous that electron flux through the cETC be downregulated through engagement of NPQ and photosynthetic control. This is necessary to ensure a balance between the rate of light-driven production of energy intermediates and their rate of consumption. In the absence of such balance, acceptor-side limitations at both photosystems could generate damaging reactive oxygen species (ROS), such as singlet oxygen at PSII and superoxide at both photosystems (Wobbe et al., 2016). The NDH pathway has a stoichiometry of 8H^+^/2e^−^ (proton-pumping complex plus cyt *b_6_f* Q-cycle) while the PGR5/PGRL1 pathway has a stoichiometry of 4H^+^/2e^−^ (Q-cycle only), meaning that the PGR5/PGRL1 pathway will be less thermodynamically constrained by an increase in *pmf*. Hence, PGR5/PGRL1 may be able to function at a higher range of ΔpH than the NDH pathway (Shikanai, 2016). This would allow enhanced engagement of the ΔpH-dependent controls over electron transport (NPQ and photosynthetic control), while at the same time continuing to use CET as a means to balance the ATP/NADPH ratio. The necessity for both of these processes tends to increase with irradiance. Another important requirement to generate the higher ΔpH, besides using PGR5/PGRL1 to maintain proton influx to the lumen, will be to reduce the conductivity of proton efflux back to the stroma through cATP synthase (Kanazawa et al., 2017; Takagi et al., 2017). It is evident that cATP synthase conductivity does change in response to metabolic conditions. For example, low stromal P_i_ (perhaps indicative of sufficient energy intermediates) is hypothesized to reduce cATP synthase conductivity, thus allowing for a higher steady-state ΔpH at any given rate of ATP synthesis (Takizawa et al., 2008). Another likely player in managing ΔpH is thylakoid membrane-localized ion transporters. For example, K^+^/H^+^ antiporter activity at low irradiance acts to reduce ΔpH while maintaining *pmf*, in this way maximizing PSII light use efficiency (Correa Galvis et al., 2020). In summary, having two distinct CET pathways allows CET to achieve two somewhat competing goals regarding energy balance – the need to increase the production ratio of ATP to NADPH (requiring dissipation of ΔpH), and the need to enhance ΔpH-dependent controls over electron transport when there is an imbalance between the generation and utilization of energy intermediates.

The ability of CET to modulate both the ATP/NADPH production ratio and ΔpH-dependent photo-protective mechanisms may be particularly challenged under conditions of rapidly fluctuating irradiance. Such conditions are common in nature, and the response of photosynthesis to such conditions is now being studied intensively (Kaiser et al., 2018; Slattery et al., 2018). Plants readily acclimate to different steady-state growth irradiances through changes in gene expression that alter the relative abundance of different components of the photosynthetic apparatus (Schöttler and Tóth, 2014). However, a continuously fluctuating irradiance will preclude such long-term acclimations, and presumably place added pressure on short-term, readily reversible regulatory mechanisms, such as those involving changes in electron and proton flux. The challenge is that optimal photosynthesis under fluctuating irradiance requires maximizing light energy absorption and use at the lower irradiance, maximizing photo-protection at the higher irradiance, and having the ability to rapidly and reversibly transition between the two states. There is strong evidence that changes in CET play an important role here. For example, while *A. thaliana pgr5* mutants can survive and grow at steady-state low or high irradiance, the mutation is seedling-lethal under fluctuating irradiance (Tikkanen et al., 2010; Suorsa et al., 2012). This primarily relates to the ability of CET to rapidly protect PSI from photo-damage due to excess electrons. Under fluctuating irradiance, CET acted to both balance the ATP/NADPH ratio, hence maintaining electron sink capacity downstream of PSI, and engage photosynthetic control, hence slowing electron flow into PSI (Kono and Terashima, 2016; Yamamoto and Shikanai, 2019). Analysis of rice mutants indicated that both pathways of CET had a role in optimizing photosynthesis under fluctuating irradiance, with the lack of either pathway resulting in significant PSI photo-damage (Yamori et al., 2016). Recent transcriptome and proteome analyses in *A. thaliana* indicate that both CET pathways increase in capacity in fluctuating irradiance (Schneider et al., 2019; Niedermaier et al., 2020).

Besides irradiance changes, other environmental factors also challenge chloroplast energy balance. Examples include changes in water status and temperature. Water deficit induces stomatal closure that, by restricting CO_2_ diffusion into the leaf, acts to slow Rubisco carboxylation and promote Rubisco oxygenation. Numerous studies suggest increased CET under such conditions, presumably to satisfy the increased ATP (relative to NADPH) demand of photorespiration relative to carbon fixation. Whether one or both CET pathways are important during water deficit remains unclear. This may depend on plant species, developmental stage, and other accompanying environmental factors (Horváth et al., 2000; Golding and Johnson, 2003; Munné-Bosch et al., 2005; Long et al., 2008; Kohzuma et al., 2009; Lehtimäki et al., 2010; Zivcak et al., 2013; Leverne and Krieger-Liszkay, 2021). The ratio between photorespiration and carbon fixation also increases with temperature. This is because increased temperature decreases the solubility of CO_2_ more than O_2_, as well as decreasing the specificity of Rubisco for CO_2_ relative to O_2_. Moderate heat stress (40–42°C) increased the rate of CET in both *A. thaliana* and tobacco (*Nicotiana tabacum*), and mutants in either CET pathway compromised photosynthesis under these conditions (Wang et al., 2006; Zhang and Sharkey, 2009; Tan et al., 2020). Besides contributing to energy balance, increased rates of CET at these high temperatures might be necessary to compensate for higher rates of thylakoid membrane leakiness to protons. In tomato (*Solanum lycopersicum*), high temperature (40°C) induced the expression of genes encoding components of both CET pathways (Lu et al., 2020). In cowpea (*Vigna unguiculata*), high temperature (45°C) substantially reduced carbon fixation while cETC activity was stimulated and dependent upon photorespiration and other unidentified electron sinks (Osei-Bonsu et al., 2021).

Several studies have linked CET to cellular amounts of H_2_O_2_. In barley (*Hordeum vulgare*), treatment of leaves with exogenous H_2_O_2_ increased amounts of NDH transcript, protein, and activity (Casano et al., 2001; Lascano et al., 2003). In tomato, a light quality-dependent systemic induction of NDH activity and a chilling-induced increase in *PGR5* gene expression were each dependent upon increases in H_2_O_2_ (Guo et al., 2016; Fang et al., 2019). In *A. thaliana*, several different photorespiration mutants, as well as several other mutants with defective expression of chloroplast proteins, all displayed both increased amounts of H_2_O_2_ and increased rates of CET (Strand et al., 2017b; Li et al., 2019). Another study clearly showed that H_2_O_2_ could rapidly increase the rate of CET in *A. thaliana* and that this activation was specific to the NDH pathway (Strand et al., 2015). The authors hypothesized that when energy imbalances cause high stromal NADPH, a lack of electron acceptor increases PSI-dependent superoxide production and conversion to H_2_O_2_. The increase in H_2_O_2_ then activates CET to correct the energy imbalance (Strand et al., 2015). In summary, there is evidence that H_2_O_2_ can increase CET through both changes in gene expression and activation at the enzyme level. Current evidence suggests that the NDH pathway is most subject to such control by H_2_O_2_. This may provide some rationale why NDH mutants show photosynthetic defects under stressful abiotic conditions known to generate oxidative stress (Shikanai, 2016). NDH mutants can also display higher amounts of H_2_O_2_ in the light (Wang et al., 2006; Sirpiö et al., 2009).

## Distinct Pathways of Mitochondrial Electron Transport to Oxygen

Malate valves function to shuttle reducing equivalents from one cell compartment to another by using isoforms of malate dehydrogenase (MDH) in different cell compartments to reversibly interconvert malate and OAA, and dicarboxylate transporters to move these metabolites between compartments (Taniguchi and Miyake, 2012; Selinski and Scheibe, 2018). To shuttle excess reducing equivalents from the stroma to cytosol during photosynthesis requires that the malate exported from the chloroplast be oxidized back to OAA, for return to the chloroplast. This malate oxidation will generate NADH, the turnover of which may then depend upon the mitochondrial electron transport chain (mETC).

A defining feature of the plant mETC is the presence of two pathways of electron flow from the ubiquinone (UQ) pool to oxygen (Figure 1B). The cytochrome (cyt) pathway consists of Complex III, cyt *c*, and Complex IV (cyt *c* oxidase). At Complex’s III and IV, electron flow is coupled to proton translocation from matrix to intermembrane space. The resulting *pmf* across the inner mitochondrial membrane (IMM) is used by the mitochondrial ATP synthase (Complex V) to generate ATP by oxidative phosphorylation. The other pathway of electron flow from UQ to oxygen consists simply of an ubiquinol oxidase termed AOX. Electron flow from UQ to AOX is not coupled to proton translocation, providing a means to relax the coupling between electron transport and ATP generation (Vanlerberghe, 2013; Del-Saz et al., 2018; Selinski et al., 2018a). In recent years, plants with increased and decreased expression of AOX have been used to examine the potential role of this pathway in supporting photosynthesis and growth (reviewed by Vanlerberghe et al., 2020). For example, if AOX respiration is necessary to balance the ATP/NADPH ratio of the chloroplast and hence prevent a stromal ATP limitation, then knockout of AOX might be expected to decrease the concentration of the ATP-dependent products of the CB cycle, ribulose 1,5-bisphosphate, and TP. Indeed, these metabolites were lower in the *A. thaliana aox1a* mutant during photosynthesis, while the amount of a non-ATP-dependent metabolite (phosphoglycerate) was unchanged relative to wild type (WT; Gandin et al., 2012). Interestingly, the mutant also contained higher amounts of the two metabolite activators of AOX1A, pyruvate, and OAA (see below).

Both modeling and experimental studies suggest that the mETC is a net sink for electrons derived from chloroplast metabolism in the light (Krömer, 1995; Buckley and Adams, 2011; Cheung et al., 2015; Shameer et al., 2019; Yamada et al., 2020; Alber and Vanlerberghe, 2021). One abundant source of electrons is a carbon reaction in the photorespiration pathway. Conversion of glycine to serine by the mitochondrial glycine decarboxylase (GDC) generates NADH. However, this does not represent a *net* source of chloroplast-derived reductant delivered to the rest of the cell since NADH that is subsequently consumed in the peroxisome by the photorespiratory enzyme hydroxypyruvate reductase stoichiometrically matches the NADH produced by GDC. [One caveat here is that some serine generated during photosynthesis may exit the photorespiratory pathway, in which case the pathway would generate some net reductant (Ros et al., 2014)]. On the other hand, stromal reductant that accumulates due to an imbalanced generation of ATP relative to NADPH by the cETC could represent a net source of chloroplast-derived reductant requiring oxidation by the mETC following export to the cytosol by the malate valve. That said, this potential source of reductant does still depend, in part, upon photorespiration. As discussed earlier, this is because photorespiratory metabolism in the chloroplast has a higher demand for ATP relative to NADPH than carbon fixation (Backhausen and Scheibe, 1999; Foyer et al., 2012; Walker et al., 2016). Hence, the chloroplast demand for ATP relative to NADPH increases as the ratio of oxygenation to carboxylation by Rubisco increases. This enhances the shortfall of ATP relative to NADPH generation by LET, hence increasing the amount of excess stromal NADPH.

The ATP produced by oxidative phosphorylation cannot readily enter the chloroplast in the light and therefore must primarily act to satisfy cytosolic ATP demand (Gardeström and Igamberdiev, 2016; Voon et al., 2018). Hence, the mETC would primarily contribute to the ATP/NADPH balance of the chloroplast by turning over chloroplast-derived reductant *via* the malate valve, which would then allow continued chloroplast electron flow coupled with photophosphorylation to boost the stromal ATP supply. Presumably, either cyt *c* oxidase or AOX could act as the sink for excess chloroplast-derived reductant, and which pathway prevails likely depends upon the cytosolic demand for ATP. When such ATP demand is low, the activity of Complex V and hence the dissipation of *pmf* across the IMM is slowed by a low amount of matrix ADP. Under these conditions, electron flow through the proton-pumping Complex’s III and IV becomes thermodynamically constrained by the increase in *pmf*. Such “adenylate control” is a major regulator of electron flow through the cyt pathway (O’Leary et al., 2019). However, the non-proton-pumping AOX is not subject to this constraint, allowing much higher rates of electron flow to oxygen when ADP is limiting (Vanlerberghe et al., 1995).

During photosynthesis, a major cytosolic demand for ATP is to support the biosynthesis of sucrose, and then the active transport of sucrose into the phloem, for delivery to sink tissues (Krömer et al., 1988; Hendrix and Grange, 1991; Krömer and Heldt, 1991; Bouma et al., 1995; Krömer, 1995; Gardeström and Igamberdiev, 2016). This demand in turn depends upon the activity of the CB cycle to provide the TP’s for sucrose biosynthesis. When carbon fixation rates are less (e.g., low irradiance, water stress), rates of sucrose synthesis and export will be less. When carbon fixation rates are greater (e.g., high irradiance, well-watered), rates of sucrose synthesis and export will be greater. Hence, the choice of whether cyt *c* oxidase or AOX consumes excess chloroplast-derived reductant may depend, in part, on net rates of carbon fixation under different environmental conditions.

**Figure 2:**
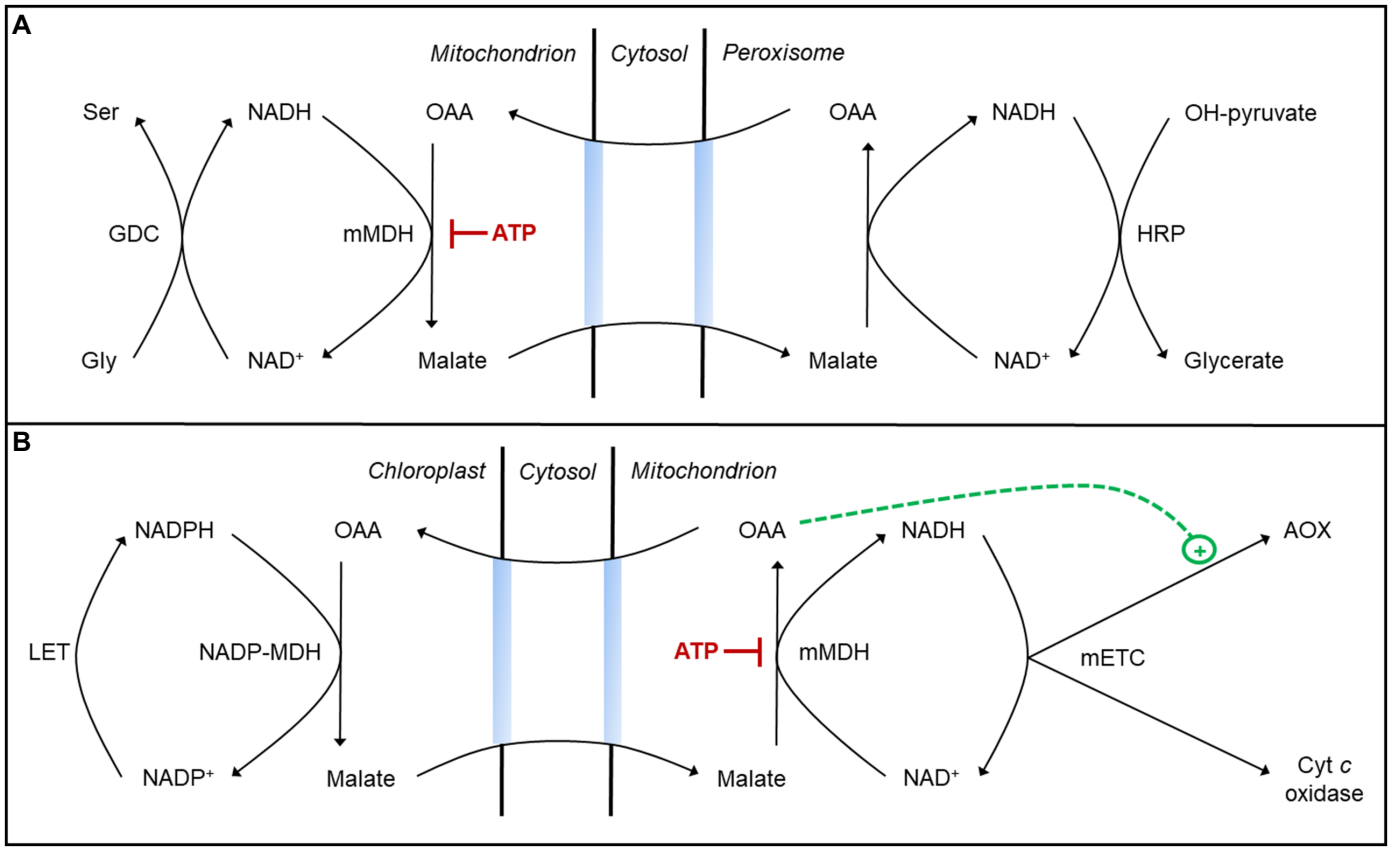
Malate valves may aid in managing the redox state of different NAD(P)H pools during photosynthesis. **(A)** Some reducing equivalents (NADH) generated during photorespiratory glycine oxidation in the mitochondrion may move to the peroxisome *via* a malate valve to support hydroxypyruvate (OH-pyruvate) reduction to glycerate (Tomaz et al., 2010; Lindén et al., 2016; Lim et al., 2020). This process requires oxaloacetic acid (OAA) reduction to malate by a mitochondrial malate dehydrogenase (mMDH), whose activity is subject to inhibition by ATP (see text for details). **(B)** Some reducing equivalents (NADPH) generated by linear electron transport (LET) in the chloroplast may move to the mitochondrion *via* a malate valve and be oxidized by the mitochondrial electron transport chain (mETC; Nunes-Nesi et al., 2005; Shameer et al., 2019; Zhao et al., 2020). This process requires malate oxidation to OAA by mMDH, whose activity is subject to inhibition by ATP. Activation of AOX by OAA may ensure that NADH and ATP amounts remain low enough to favor this malate oxidation (see text for details).

A key component of the chloroplast malate valve is an NADP-dependent MDH (NADP-MDH) in the stroma that couples NADPH oxidation with the reduction of OAA to malate (Taniguchi and Miyake, 2012). The malate produced then exits the chloroplast and is oxidized back to OAA in the cytosol or other cell compartment by NAD-dependent MDH isoforms, producing NADH. The activity of NADP-MDH is subject to tight thiol-based redox regulation to ensure that the valve is only active when the stromal NADPH/NADP^+^ ratio is high (Selinski and Scheibe, 2018). Interestingly, mitochondrial MDH (mMDH) is not redox-regulated but rather is regulated by adenylates acting as direct effectors of enzyme activity. In *A. thaliana*, mMDH1 activity (either malate oxidation or OAA reduction) is inhibited by an increase in the ATP/ADP ratio (Yoshida and Hisabori, 2016b). This result is consistent with earlier studies in mung bean (Asahi and Nishimura, 1973; Tobin and Givan, 1984). Hence, a high matrix ATP/ADP ratio could compromise the cell’s ability to manage the redox state of different NAD(P)H pools. For example, if mitochondrial OAA reduction to malate slows, it could compromise the shuttling from mitochondrion to peroxisome of reducing equivalents arising from photorespiratory glycine oxidation (Tomaz et al., 2010; Lindén et al., 2016; Lim et al., 2020; Figure 2A). On the other hand, if mitochondrial malate oxidation to OAA slows, it could compromise the shuttling of reducing equivalents from the chloroplast to the mETC (Nunes-Nesi et al., 2005; Shameer et al., 2019; Zhao et al., 2020; Figure 2B). Hence, preventing either of these scenarios may in some cases depend upon a shift in the mETC from the cyt pathway to AOX in order to lower the ATP yield and hence ensure sufficient mMDH activity to manage the redox state of different NAD(P)H pools during photosynthesis.

Specific metabolites act as direct effectors of AOX activity, allowing for rapid and reversible changes in the partitioning of electrons between the cyt pathway and AOX in response to metabolic conditions within the mitochondrial matrix (Millar et al., 1993). Across plant species, pyruvate is a potent activator of multiple AOX isoforms (Millar et al., 1993; Vanlerberghe et al., 1995; Selinski et al., 2018b). A comprehensive study of AOX effectors in *A. thaliana* has shown that the AOX1A isoform is activated by both pyruvate and OAA (Selinski et al., 2018b). Pyruvate is the product of malate oxidation by NAD-dependent malic enzyme (NAD-ME), while OAA is the product of malate oxidation by mMDH. High matrix NADH favors NAD-ME over mMDH activity, since the equilibrium of the mMDH reaction strongly favors OAA reduction (Tobin et al., 1980). If malate is being imported into the mitochondrion as a means to shuttle reducing equivalents from the chloroplast to mETC, then mMDH activity is needed to generate the OAA for return to the chloroplast. AOX1A activation by OAA could be important to ensure that NADH amounts remain low and favor malate oxidation by mMDH, even when ATP/ADP ratios might otherwise constrain NADH oxidation by the cyt pathway (Selinski et al., 2018b; Figure 2B). This may provide some rationale why other AOX isoforms not activated by OAA appear unable to compensate for AOX1A in optimizing photosynthesis (Strodtkötter et al., 2009).

The stimulation of AOX activity by pyruvate and other metabolic activators first requires that the AOX dimer be present in its “reduced” form, where a regulatory disulfide bond between the two monomers is reduced to its component sulfhydryls (Umbach and Siedow, 1993; Vanlerberghe et al., 1995). In isolated mitochondria, this reduction is initiated by the oxidation of specific tricarboxylic acid cycle intermediates, which presumably provides NADPH used by a mitochondrial thioredoxin system to catalyze the reduction (Vanlerberghe et al., 1995; Gelhaye et al., 2004). However, the physiological relevance of this oxidation and reduction for rapid and reversible changes in AOX activity remains uncertain since the reduced form of AOX is usually shown to dominate *in vivo*. This suggests that short-term biochemical control of AOX activity will depend primarily upon the concentration of metabolic effectors. However, in *Alocasia odora*, a shade species, there was a striking effect of growth irradiance on AOX redox status (Noguchi et al., 2005). While the total amount of AOX protein was similar between high irradiance-grown [photosynthetic photon flux density of 490μmolm^−2^ s^−1^ (490 PPFD)] and low irradiance-grown (20 PPFD) plants, the AOX dimer was predominantly in its reduced active form in the former, but oxidized inactive form in the later. This might allow the low irradiance-grown plants to maintain the low respiration rate typical of shade plants, while also allowing for a rapid increase in AOX activity following sudden exposure to higher irradiance (Noguchi et al., 2005).

Higher amounts of photorespiration (relative to carbon fixation) increases the chloroplast demand for ATP relative to NADPH, while at the same time reducing cytosolic ATP demand for sucrose synthesis and export, due to a relative slowing of carbon gain. Hence, as photorespiration increases, one might expect a shift toward more AOX respiration to oxidize excess reductant. In *A. thaliana*, the glycine to serine ratio gradually increases with increased temperature, presumably since higher temperatures promote photorespiration (Li et al., 2020). In an *aox1a* mutant, the glycine to serine ratio was similar to WT at lower temperatures (4–12°C) but higher than WT at higher temperatures (23–35°C; Li et al., 2020). This suggests that GDC was inhibited in *aox1a* at higher temperatures, likely by high matrix NADH (Pascal et al., 1990; Bykova et al., 2014). Hence, as photorespiration becomes more active, AOX activity is necessary for redox balance, and the cyt pathway apparently cannot compensate for the lack of AOX, perhaps due to an insufficient demand for cytosolic ATP. Similarly, another study showed that photosynthesis in an *A. thaliana aox1a* mutant was only disrupted under photorespiratory conditions (Zhang et al., 2017).

When the mETC is using AOX to facilitate electron movement from NADH to oxygen, only one site of proton translocation (Complex I) is active, hence lowering the ATP yield associated with electron flow. What happens if this ATP yield is still too high to allow sufficient turnover of reductant by the mitochondrion during photosynthesis? In this case, two other mETC components could potentially further boost the capacity of the mitochondrion to act as electron sink. First, plant mitochondria contain a family of alternative NAD(P)H dehydrogenases that, unlike Complex I, are not proton-pumping (Rasmusson et al., 2020; Figure 1B). Hence, if their activity was combined with AOX, electron flow from NAD(P)H to oxygen would involve no sites of proton translocation to support oxidative phosphorylation. Second, plant mitochondria have uncoupling proteins (UCPs) that are able to dissipate the proton gradient across the IMM, hence uncoupling electron transport from oxidative phosphorylation (Barreto et al., 2020; Figure 1B). In *A. thaliana* and other species, there is co-expression of the genes encoding AOX1A and the alternative dehydrogenase NDB2 (Clifton et al., 2005; Yoshida and Noguchi, 2009; Wanniarachchi et al., 2018; Sweetman et al., 2020). NDB2 is the major external NADH dehydrogenase, meaning that it oxidizes NADH on the external (i.e., cytosolic) side of the IMM. An inter-dependence between NDB2 and AOX1A activities exists that could relate to redox poising of the UQ pool (Sweetman et al., 2019). However, whether alternative dehydrogenases have a role in supporting photosynthesis remains unclear since there have been relatively few studies of plants with altered expression of these dehydrogenases (Sweetman et al., 2019). More studies have examined the potential role of UCP’s during photosynthesis. GDC activity was restricted in an *A. thaliana ucp1* mutant that, interestingly, was also deficient in AOX protein (Sweetlove et al., 2006). Another interesting observation was that UCP1 overexpression in tobacco resulted in an increased abundance of transcripts encoding several major cETC components, but not mETC components (Laitz et al., 2015). In another study, inhibition of mitochondrial oxidative phosphorylation in the light with oligomycin resulted in a strong induction of both UCP and cATP synthase that was then able to maintain cETC activity and cell viability (Alber and Vanlerberghe, 2021). These studies suggest a connection between mitochondrial UCP’s and photosynthesis that deserves further study.

## Interaction of the Different Chloroplast Cet and Mitochondrial Electron Transport Pathways

Evidence in the literature suggests that CET and the mETC may compensate for each other’s activity, perhaps signifying some complementarity of function. Following growth at low irradiance (40 PPFD), both *pgr5* and *crr2-2* (NDH) mutants of *A. thaliana* had a near 2-fold greater amount of AOX protein, compared to WT (Yoshida et al., 2007). Following an 8h high irradiance (320 PPFD) treatment of these plants, the AOX amount was now similar in WT and *crr2-2*, primarily due to an approximately 2-fold increase of AOX amount in the WT, with little change in *crr2-2*. On the other hand, the high irradiance treatment doubled the AOX amount in the *pgr5* mutant, such that these plants now maintained a much higher AOX amount than WT or *crr2-2* plants (Yoshida et al., 2007). These results suggest that both pathways of CET are consequential at low irradiance, with knockdown of either resulting in a compensatory increase in AOX amount. However, following the shift to high irradiance, only the loss of PGR5 required a further compensatory increase in AOX, suggesting that the NDH pathway was now less consequential than the PGR5/PGRL1 pathway. Further, the increase of AOX in the WT plants in response to the irradiance shift suggests that both PGR5 and AOX are beneficial under these conditions. Like the CET pathways, AOX could provide a means to adjust the ATP/NADPH ratio of the chloroplast (by consuming chloroplast-derived reductant) and/or bolster the ΔpH-dependent controls over cETC activity by promoting LET. Cyt *c* oxidase could equally fulfill these roles but only if its activity was not being constrained by rates of cytosolic ATP turnover, the demand for which may not increase, at least in the shorter-term, following transfer to higher irradiance. Another study found that a double mutant lacking both CET pathways not only had a 2-fold higher AOX protein content but also a significant decline in cyt *c* oxidase (subunit II) protein. This was evident even at a low growth irradiance (80 PPFD), indicating a fundamental shift in the composition of the respiratory chain (Florez-Sarasa et al., 2016).

Other studies also imply complementary roles of the chloroplast PGR5/PGRL1 and mitochondrial AOX pathways. In *C. reinhardtii,* a *pgrl1* mutant displayed normal photosynthesis and growth under a wide range of irradiance and CO_2_ concentration. This was due in part to changes in respiration in the mutant, including increased amounts of AOX (Dang et al., 2014). However, growth of the mutant became compromised in fluctuating light, suggesting that respiration was unable to compensate for the lack of CET under those conditions. To our knowledge, it is unknown whether AOX respiration has a role in C_3_ plants under fluctuating light conditions, so this should be a priority for future study. Interestingly, photosynthetic performance was compromised under fluctuating light in plants with a mutated chloroplast NADP-MDH lacking redox regulation (Yokochi et al., 2021). Further, the expression and protein amount of mitochondrial GDC and other photorespiratory enzymes increased in fluctuating light (Schneider et al., 2019; Niedermaier et al., 2020). This suggests that the high light phase of fluctuating light can result in leaf CO_2_ depletions that promote photorespiration, which could then necessitate an increased use of the malate valve and mitochondrial functions to balance the stromal ATP/NADPH ratio. During the high light phase of fluctuating light, increases in carbon fixation and sucrose synthesis likely lag behind increases in cETC activity. Hence, there would be a transient increased demand on the mitochondrion to oxidize excess NADPH without a corresponding increase in ATP demand for sucrose synthesis. These conditions could necessitate the use of AOX as the mitochondrial electron sink.

Studies have also examined an *A. thaliana aox1a*/*pgr5* double mutant (Yoshida et al., 2011a; Jiang et al., 2019). The double mutant grew much more poorly than either single mutant, particularly at early growth stages and higher growth irradiances (note that of the single mutants, only *pgr5* showed compromised growth relative to WT; Yoshida et al., 2011a). The growth phenotype of the double mutant may be at least partly due to a reduced rate of carbon fixation at early growth stages (Jiang et al., 2019). These studies emphasize that, even with a functional chloroplast NDH and mitochondrial cyt pathway in place, the lack of both lower-ATP yielding pathways (PGR5/PGRL1 and AOX) can severely hinder plant performance relative to the lack of either one alone.

A potential consequence of having excess chloroplast reductant oxidized by the mETC is that it moves the burden of ROS generation from the cETC to the mETC (Zhao et al., 2020). If only cyt *c* oxidase was present, then the extent to which turnover of chloroplast reductant generated mitochondrial ROS would depend upon the demand for mitochondrial-generated ATP, such as to support sucrose synthesis. Low ATP demand would increase the reduction state of mETC components, hence enhancing ROS generation (Møller, 2001). Instead, the presence of AOX will moderate the reduction state of mETC components when ATP demand is low. Hence, utilizing AOX, rather than the cyt pathway, to manage excess chloroplast reductant should be of particular importance when the demand for mitochondrial-generated ATP is low. This would include conditions, such as water deficit, that slow CB cycle activity by restricting CO_2_ supply. This has multiple potential impacts. First, it exaggerates the imbalance between energy generation in the thylakoids and energy use in the stroma. Second, it exaggerates the ATP/NADPH imbalance in the chloroplast by promoting photorespiration. Third, since carbon fixation is restricted, it reduces the cytosolic ATP demand for sucrose synthesis and export. Indeed, some studies have shown that, under water deficit, oxidative damage can occur earlier in leaf mitochondria than chloroplasts (Bartoli et al., 2004; Dahal and Vanlerberghe, 2017).

In tobacco experiencing water deficit, oxidative damage (protein carbonylation amount) was enhanced in *AOX1A* knockdowns in both the mitochondrion and chloroplast, suggesting that the AOX pathway was a necessary electron sink and acted to prevent ROS generation in both organelles (Dahal and Vanlerberghe, 2017). This occurred despite the knockdowns displaying higher rates of CET than WT under water deficit conditions (Dahal et al., 2014; Dahal and Vanlerberghe, 2018a). The protein oxidative damage correlated closely with losses of PSII function in the chloroplast and cyt *c* oxidase function in the mitochondrion. On the other hand, AOX overexpression reduced oxidative damage and preserved the organelle functions relative to WT plants (Dahal and Vanlerberghe, 2017). Other studies also report increased rates of CET in the absence of AOX. In broad bean (*Vicia faba*), chemical inhibition of AOX decreased the ratio of PSII to PSI operating efficiency, particularly at low measurement irradiance, suggestive of increased CET (Yoshida et al., 2006). Similar results were seen in the *A. thaliana aox1a* mutant, but at high rather than low measurement irradiance (Yoshida et al., 2011a).

Rates of sucrose synthesis and export, and hence demand for cytosolic ATP, are expected to increase when plants are grown at elevated (i.e., above ambient) atmospheric concentrations of CO_2_ (ECO_2_; Paul and Foyer, 2001; Long et al., 2004). These conditions should also suppress photorespiration, hence easing the ATP/NADPH imbalance associated with LET. Nonetheless, tobacco showed a preferential increase in AOX transcript and protein (relative to cyt *c* oxidase) under such conditions suggesting a role for AOX other than maintaining energy balance (Chadee and Vanlerberghe, 2020). Interestingly, the increase in AOX at ECO_2_ was accompanied by the increased expression of a sugar-responsive gene encoding a chloroplast glucose-6-phosphate/phosphate translocator (GPT; Chadee and Vanlerberghe, 2020). Further, tobacco AOX1A knockdowns grown at ECO_2_ had higher amounts of leaf starch and sucrose than WT plants (Dahal and Vanlerberghe, 2018b). These results suggest that, in some circumstances, leaf AOX respiration may provide a means to consume excess carbohydrate that accumulates due to an imbalance between photosynthetic activity and sink carbohydrate demand. Such carbon imbalances can result in both short-term bottlenecks in photosynthetic activity and long-term declines in photosynthetic capacity (Paul and Foyer, 2001; Fabre et al., 2019; McClain and Sharkey, 2019). Well-watered tobacco plants grown at ECO_2_ displayed slightly lower rates of CET than plants grown at ambient CO_2_. Nonetheless, knockdown of AOX had no effect on the rate of CET in ambient-grown plants, but did increase the rate of CET in the plants grown at ECO_2_, compared to WT (Dahal and Vanlerberghe, 2018b). This suggests some compensatory function of CET in the AOX knockdowns suffering carbon imbalance. Increased GPT expression appears to be a common response to growth at ECO_2_ across species (Li et al., 2006; Leakey et al., 2009; Chadee and Vanlerberghe, 2020). It presumably allows some flux of glucose-6-phosphate back into the chloroplast during photosynthesis, perhaps as part of a response to carbon imbalance. This flux could support additional starch synthesis (Dyson et al., 2015) or potentially feed carbon back into the CB cycle *via* a glucose-6-phosphate shunt (Sharkey and Weise, 2016; Preiser et al., 2019; Weise et al., 2019). This shunt can be a dominating contributor to CO_2_ release in the light (Xu et al., 2021) and consumes ATP (Sharkey and Weise, 2016). The increase in CET in the AOX1A knockdowns suffering carbon imbalance might be to compensate for ATP consumption by such shunt activity, but this hypothesis awaits further investigation.

Interestingly, at least three *A. thaliana* transcriptome studies have identified both AOX and GPT genes as among those that rapidly increase in expression following a shift to higher irradiance (Kleine et al., 2007; Crisp et al., 2017; Huang et al., 2019). In one study, *AOX1A*, *AOX1C*, *AOX1D*, and *GPT2* were among a core set of 250 genes differentially regulated at all six time points (between 30min and 72h) after transfer from a growth irradiance of 60 PPFD to a treatment irradiance of 1,200 PPFD (Huang et al., 2019). At each time point, the *AOX* and *GPT2* transcripts were increased in abundance, while most photosynthesis-related gene transcripts either declined or remained unchanged. The transcript of the alternative dehydrogenase NDB2 was also increased at most time points. Carbon fixation may have been inhibited during the 72h high irradiance treatment, at least based on the clear suppression of growth compared to plants maintained at the low irradiance (Huang et al., 2019). This perhaps leaves an open question of whether the AOX and GPT2 changes at high irradiance were occurring primarily in response to a carbon or energy imbalance.

PGR5/PGRL1-dependent CET is likely dependent upon additional protein components not yet identified. Nonetheless, the pathway likely depends upon far fewer protein components than NDH, which is the largest complex in the cETC. Similarly, the AOX pathway of electron flux from UQ to oxygen is simple in terms of protein composition relative to the cyt pathway components involved in electron flux from UQ to oxygen (Complex III+cyt *c*+Complex IV). The relatively simple composition of the PGR5/PGRL1 and AOX pathways may allow the plant to increase the capacity of these pathways relatively rapidly, and with relatively little investment of energy for biosynthesis. If this is the case, acclimation to acute environmental changes may preferentially rely upon rapid and low cost adjustment of PGR5/PGRL1 and AOX pathway capacities, while chronic environmental changes might rely preferentially upon slower and more costly adjustments in the NDH and cyt pathway capacities. For example, in *A. thaliana*, plants grown long term at high irradiance had higher amounts of cyt *c* oxidase subunit II protein than plants grown at low irradiance, while the amount of AOX protein was similar between the two growth conditions. On the other hand, plants shifted short term to higher irradiance displayed a rapid increase in AOX protein but no change in cyt *c* oxidase (Yoshida et al., 2011b). To our knowledge, no comparable study has examined the response of both CET pathways to both long-term and short-term changes in irradiance, so this would be an interesting subject to investigate. In pea, AOX capacity and protein amount increased within 10min of a high irradiance treatment (Dinakar et al., 2010).

The pathways of electron transport that may be preferentially responding to acute environmental changes (PGR5/PGRL1 and AOX) are also those with the lower ATP yield. This would inherently increase the flexibility of the metabolic network under changing conditions by loosening the coupling between electron transport and ATP generation. The AOX increase would rapidly enhance the capacity of the mitochondrion to act as an electron sink, while the PGR5/PGRL1 increase would rapidly enhance the capacity of CET to build up the thylakoid ΔpH. These changes would also act to minimize ROS generation in both organelles.

## Conclusion

We hypothesize that chloroplast PGR5/PGRL1 and mitochondrial AOX represent stress-inducible electron transport pathways that, owing to their lower ATP yield (compared to the chloroplast NDH and mitochondrial cyt pathway, respectively), rapidly increase the flexibility of metabolism within their respective organelles. Stress conditions (e.g., water stress and temperature extremes) typically reduce energy demands for carbon fixation and sucrose synthesis/export. A shift toward AOX would maintain the electron sink capacity of the mitochondrion, despite lower cytosolic ATP demand. Meanwhile, a shift toward PGR5/PGRL1 would enhance chloroplast photo-protection, in the face of overall lower energy demands for carbon fixation. This would protect both organelles from excessive ROS production and oxidative damage during stress. It may be relevant to identify physiological or biochemical constraints that normally limit the function of one or the other pathway under particular environmental conditions, hence necessitating an increased contribution from the alternate organelle to maintain energy and carbon balance during photosynthesis. There may also be more specific means to coordinate the activity of the two pathways. Photorespiratory H_2_O_2_ has been hypothesized to coordinate CET and AOX activities, but the details of this remain uncertain (Sunil et al., 2019).

## Author Contributions

All authors contributed insight toward, and commented on, a draft version of the manuscript prepared by GV. All authors approved the submitted version of the manuscript.

## Conflict of Interest

The authors declare that the research was conducted in the absence of any commercial or financial relationships that could be construed as a potential conflict of interest.

## Publisher’s Note

All claims expressed in this article are solely those of the authors and do not necessarily represent those of their affiliated organizations, or those of the publisher, the editors and the reviewers. Any product that may be evaluated in this article, or claim that may be made by its manufacturer, is not guaranteed or endorsed by the publisher.
